# A New Pronouncing Dictionary of Medicine

**Published:** 1892-09

**Authors:** 


A NEW PRONOUNCING DICTIONARY OF MEDICINE. Being a voluminous and exhaustive hand-book of Medical and Scientific Terminology, with Phonetic Pronunciation, Accentuation, Etymology, etc. By John M. Keating, M. D., LL. D., Fellow of the College of Physicians of Philadelphia; Vice-President of the American Pædiatric Society; Ex-President of the Association of Life Insurance Medical Directors; formerly Visiting Obstetrician to the Philadelphia Hospital (Blockley), and Lecturer on the Diseases of Women and Children; Consulting Physician for the Diseases of Women, St. Agnes' Hospital, Philadelphia; Gynæcologist to St. Joseph's Hospital, Philadelphia; Editor "Cyclopædia of the Diseases of Children," etc., and Henry Hamilton, author of "A new Translation of Virgil's Æneid into English Rhyme;" Co-Author of "Saunders' Medical Lexicon," etc. With the Collaboration of J. Chalmers Da Costa, M. D., and Frederick A. Packard, M. D. With an appendix containing important tables of Bacilli, Miccrococci, Leucomaines, Ptomaines, Drugs, and Materials used in Antiseptic Surgery; Poisons and their Antidotes; Weights and Measures; Thermometric Scales; New Officinal and Unofficinal Drugs, etc., etc. Philadelphia: W. B. Saunders, 913 Walnut Street. 1892. Price, cloth, $5.00; sheep, $6.00 net.

This work of something like eight hundred and twenty pages begins with an introductory notice in which the learned authors tell us that the matter of *pronunciation* has had their first attention, on account of the diversity of teaching regarding this matter in the medical colleges. In order to be accurate, they have consulted the men of learning in the different universities of America and introduce the replies they have received from them. The answers of these Professors of Latin and Greek are very interesting.

Following the very lucid introduction we have a "Table of Medical Abbreviations" that is indeed a marvel of simplicity and brevity, and yet complete. Then we have a table of suffixes and prefixes from a to z.

The Pronouncing Medical Dictionary now follows. First we find the proper word with correct pronunciation. Now the Greek or Latin derivation is given, and lastly the meaning of the whole in excellent English. Everything is very complete. Hundreds of new words have been added and obsolete terms omitted. Indeed, the whole is a grand improvement over any other dictionary in the market. But why say more? The work must be seen to be appreciated. It is the best work on the subject. The paper, printing, and binding are superb. No medical student and practitioner can afford to be without the great Pronouncing Dictionary of Medicine by Professor Keating.

